# Sleeping with Hippocampal Damage

**DOI:** 10.1016/j.cub.2019.11.072

**Published:** 2020-02-03

**Authors:** Goffredina Spanò, Frederik D. Weber, Gloria Pizzamiglio, Cornelia McCormick, Thomas D. Miller, Clive R. Rosenthal, Jamie O. Edgin, Eleanor A. Maguire

**Affiliations:** 1 Wellcome Centre for Human Neuroimaging, UCL Queen Square Institute of Neurology, University College London, London WC1N 3AR, UK; 2 Donders Institute for Brain, Cognition and Behaviour, Radboud University Medical Centre, Nijmegen 6525 EN, the Netherlands; 3 Department of Neurodegenerative Diseases and Geriatric Psychiatry, University Hospital Bonn, Bonn 53127, Germany; 4 Department of Neurology, Royal Free Hospital, London NW3 2QG, UK; 5 Nuffield Department of Clinical Neurosciences, University of Oxford, Oxford OX3 9DU, UK; 6 Department of Psychology, University of Arizona, Tucson, AZ 85721, USA

**Keywords:** sleep, hippocampus, amnesia, polysomnography, slow-wave sleep, fast spindles, slow oscillations, ripples, episodic memory, memory consolidation

## Abstract

The hippocampus plays a critical role in sleep-related memory processes [1, 2, 3], but it is unclear which specific sleep features are dependent upon this brain structure. The examination of sleep physiology in patients with focal bilateral hippocampal damage and amnesia could supply important evidence regarding these links. However, there is a dearth of such studies, despite these patients providing compelling insights into awake cognition [4, 5]. Here, we sought to identify the contribution of the hippocampus to the sleep phenotype by characterizing sleep via comprehensive qualitative and quantitative analyses in memory-impaired patients with selective bilateral hippocampal damage and matched control participants using in-home polysomnography on 4 nights. We found that, compared to control participants, patients had significantly reduced slow-wave sleep—likely due to decreased density of slow waves—as well as slow-wave activity. In contrast, slow and fast spindles were indistinguishable from those of control participants. Moreover, patients expressed slow oscillations (SOs), and SO-fast spindle coupling was observed. However, on closer scrutiny, we noted that the timing of spindles within the SO cycle was delayed in the patients. The shift of patients’ spindles into the later phase of the up-state within the SO cycle may indicate a mismatch in timing across the SO-spindle-ripple events that are associated with memory consolidation [6, 7]. The substantial effect of selective bilateral hippocampal damage on large-scale oscillatory activity in the cortex suggests that, as with awake cognition, the hippocampus plays a significant role in sleep physiology, which may, in turn, be necessary for efficacious episodic memory.

## Highlights

• We examined sleep physiology in patients with focal bilateral hippocampal damage
• General features of sleep physiology and quality were intact in patients
• Patients exhibited significantly reduced SWS and decreased SWA in N2 sleep
• Fast spindles also occurred later in the slow oscillation cycle in patients

## Teaser

By examining sleep physiology in patients with focal bilateral hippocampal damage, Spanò et al. show that hippocampal integrity seems necessary for slow-wave sleep regulation and for fine-tuning the timing of slow oscillation-fast spindle coupling, both of which are held to be necessary for memory consolidation during sleep.

## Results

We examined sleep architecture in four patients (all right-handed males; mean age 58.25 years ± SD 20.82) with focal lesions to the hippocampus bilaterally and with a significant episodic memory deficit for their personal past experiences (STAR Methods; Figure S1; Tables S1 and S2). Patients were matched to ten healthy control participants (all right-handed males; mean age 59.2 years ± 15.89) on demographic factors (age, gender, body mass index, and non-verbal IQ; STAR Methods). We conducted an in-depth examination of their sleep phenotype using a range of complementary approaches (STAR Methods). These included standardized questionnaires assessing habitual sleep habits over the last month (The Pittsburgh Sleep Quality Index) [8], level of daytime sleepiness (The Epworth Sleepiness Scale) [9], and chronotype—whether someone is a “morning” or an “evening” type of person (The Morningness-Eveningness Questionnaire) [10]; WatchPAT-200 (Itamar Medical, Caesarea, Israel), a diagnostic device that detects obstructive sleep apnea using peripheral arterial tone [11]; Actiwatch 2 (Phillips Respironics Mini-Mitter), a non-invasive method of monitoring human rest/activity cycles, over 7 consecutive days and nights; and polysomnography (PSG; EASYCAP Brain Products, Gilching, Germany), which measures neural activity via scalp electroencephalogram (EEG) and other bioparameters, such as eye movements (electrooculography [EOG]), muscle activity (electromyogram [EMG]), and heart rate (electrooculography [ECG]) during sleep.

Often PSG studies are performed in a sleep laboratory, but this can adversely affect sleep quality. We therefore recorded PSG in participants’ own homes on 4 nights. During the first habituation night, participants were familiarized with the PSG equipment and procedure, and WatchPAT data were collected. PSG data from this night were not included in the analyses. We conducted the analyses of PSG recordings using 3 subsequent nights, separated on average by 18 days ± 7, thus allowing for an assessment of sleep architecture consistency, and ensuring that results were not driven by an individual night.

It is important to note that the small sample of these rare patients might potentially mask group differences. For this reason, along with the results of the statistical tests and concomitant p values, we have also included confidence intervals and effect sizes, performed additional bootstrapped analyses (sampling the dataset with resampling; 1,000 iterations), and used several different methods for data analysis (e.g., visual sleep staging; an automatic slow-wave detection algorithm) to aid interpretation.

### Sleep Quality

We first examined sleep quality. Table 1 shows the summary data and statistical analyses. The patient and control groups did not differ on the questionnaire measures of general quality and patterns of sleep, level of daytime sleepiness, and chronotype. There was also no difference between the groups on objective measures of sleep quality. Equivalent outcomes included WatchPAT’s apnea-hypopnea index, the Actiwatch measures of total sleep time, sleep efficiency (the percentage of time spent in bed sleeping; mean >80% in both groups), and sleep fragmentation (the percentage of sleep considered to be restless due to consistent physical movement). These results show that general features of sleep quality are unlikely to be mediated by the hippocampus.

**Table 1:** Sleep Characteristics of the Patients and Control Participants

	HPC		CTL		U	ES	p Value
	M (SD)	95% CI	M	95% CI			
**Sleep Questionnaires**							

PSQI	3.50 (1.00)	[1.91 to 5.09]	4.90 (3.31)	[2.53 to 7.27]	16.5	0.27	0.604
ESS	9.25 (8.34)	[−4.02 to 22.52]	5.00 (3.59)	[2.43 to 7.57]	13.5	0.51	0.350
MEQ	53.50 (5.8)	[44.27 to 62.73]	58.60 (6.06)	[54.27 to 62.93]	11.5	0.68	0.228

**WatchPAT**							

Apnea-hypopnea index	17.58 (15.21)	[−6.63 to 41.78]	10.13 (6.66)	[5.37 to 14.89]	13.0	0.55	0.322

**Actigraphy (across 7 Nights)**							

Sleep efficiency (%)	83.44 (9.43)	[68.42 to 98.45]	89.25 (2.83)	[87.22 to 91.27]	10.0	0.82	0.157
Total sleep time (min)	413.79 (88.33)	[273.24 to 554.34]	420.49 (32.97)	[396.97 to 444]	17.0	0.23	0.671
Fragmentation index	28.50 (13.01)	[7.79 to 49.2]	18.48 (6.23)	[14.02 to 22.94]	14.0	0.47	0.396
Night-to-night variability	9.82 (4.90)	[2.03 to 17.61]	7.42 (2.36)	[5.73 to 9.11]	15.0	0.39	0.480
Bedtime	23:50 (01:00)	[22:14 to 01:26]	23:30 (00:32)	[23:07 to 23:53]	18.0	0.15	0.777
Midpoint	3:41 (0:35)	[2:45 to 4:37]	3:17 (0:33)	[2:53 to 3:40]	11.0	0.72	0.203

**Sleep Macroarchitecture**							

Total sleep time (min)	303.71 (70.91)	[190.87 to 416.55]	326.98 (59.57)	[284.37 to 369.6]	14.0	0.47	0.396
Sleep efficiency (%)	65.83 (12.33)	[46.21to 85.45]	71.22 (11.08)	[63.29 to 79.14]	15.0	0.39	0.480
Latency to sleep onset (min)	22.15 (10.52)	[5.41to 38.88]	14.82 (8.66)	[8.62 to 21.01]	8.0	1.02	0.090
Latency to REM (min)	114.28 (37.44)	[21.27 to 207.29]	84.02 (27.62)	[64.26 to 103.77]	6.0	1.25	0.128
Wake after sleep onset (min)	132.27 (61.62)	[34.22 to 230.32]	116.22 (46.17)	[83.19 to 149.24]	17.5	0.19	0.723
NREM stage 1 (%)	25.35 (20.03)	[−6.53 to 57.22]	17.86 (5.77)	[13.73 to 21.99]	19.0	0.08	0.888
NREM stage 1 (min)	66.46 (43.88)	[−3.37 to 136.29]	55.6 (11.88)	[47.1 to 64.1]	19.0	0.08	0.888
NREM stage 2 (%)	57.49 (14.75)	[34.02 to 80.96]	55.8 (4.27)	[52.74 to 58.86]	14.0	0.47	0.396
NREM stage 2 (min)	179.69 (68.16)	[71.24 to288.14]	183.27 (40.44)	[154.33 to 212.2]	18.0	0.15	0.777
NREM SWS (%)	0.33 (0.44)	[−0.36 to 1.03]	5.39 (4.57)	[2.12 to 8.65]	3.0	1.68	0.016^a^
NREM SWS (min)	1.31 (1.93)	[−1.77 to 4.39]	18.02 (16.76)	[6.02 to 30.01]	3.0	1.68	0.016^a^
REM sleep (%)	16.83 (14.04)	[−5.51 to 39.18]	20.97 (4.47)	[17.77 to 24.17]	12.0	0.63	0.258
REM sleep (min)	56.25 (56.71)	[−33.98 to 146.48]	69.92 (19.46)	[56 to 83.83]	12.0	0.63	0.258

**Sleep Stability and Fragmentation**							

Arousal index score events/h	31.01 (26.24)	[−10.74 to 72.77]	21.42 (6.94)	[16.46 to 26.39]	17.0	0.23	0.671
Awakening index score events/h	8.54 (2.60)	[4.4 to 12.68]	9.27 (3.03)	[7.11 to 11.44]	17.0	0.23	0.671
State transitions/h	43.73 (21.74)	[9.14 to 78.32]	45.73 (11.79)	[37.3 to 54.17]	19.0	0.08	0.888
Functional uncertainty (TFU/TST)	0.16 (0.10)	[0 to 0.33]	0.17 (0.07)	[0.12 to 0.23]	18.0	0.15	0.777
Wake-sleep transitions (min)	0.20 (0.10)	[0.04 to 0.37]	0.21 (0.09)	[0.15 to 0.28]	20.0	0.00	1.000
Stability NREM (min)	1.89 (0.15)	[1.66 to 2.13]	1.77 (0.09)	[1.7 to 1.83]	10.0	0.82	0.157
Stability REM (min)	1.75 (0.19)	[1.27 to 2.23]	1.68 (0.08)	[1.63 to 1.74]	9.0	0.91	0.310

Sleep macroarchitecture and sleep stability and fragmentation measures are averaged over 3 nights. For one patient, the average was based on 2 PSG nights due to artifacts in the recording of the other night. p values relate to between-group non-parametric Mann-Whitney U tests. See Table S3 for the results of the bootstrapped analysis. Note that one patient had severe obstructive sleep apnea. When he and his matched controls were removed from the analyses, the results were unchanged—see STAR Methods and Tables S4 and S5. 95% CI, 95% confidence interval; CTL, control participants; ES, effect size; ESS, Epworth Sleepiness Scale; HPC, hippocampal-damaged patients; M, mean; MEQ, Morningness-Eveningness Questionnaire; min, minute; NREM, non-rapid eye movement sleep; PSQI, Pittsburgh Sleep Quality Index; REM, rapid eye movement sleep; SD, standard deviation; SWS, slow-wave sleep; TFU, total functional uncertainty; TST, total sleep time.

a Significant differences

### Sleep Macroarchitecture

We further interrogated total sleep time, sleep efficiency, latency to sleep onset, and periods of wakefulness occurring after sleep onset using the PSG data (see Table 1 for summary data and statistical analyses; STAR Methods). The results aligned with the findings from the Actiwatch data, with no significant differences apparent between the two groups.

**Figure 1:**
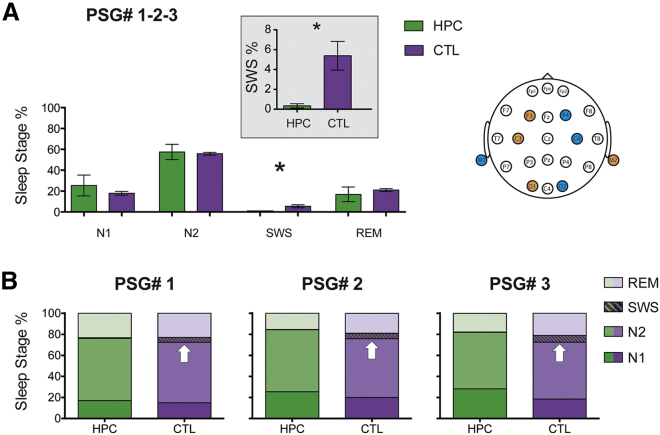
Sleep Stage Comparisons between the Patient and Control Participants (A) To assess potential differences in sleep architecture between the patients and control participants, we averaged sleep studies conducted on 3 separate nights (1-2-3; means and SEMs are shown). CTL, control participants; HPC, hippocampal-damaged patients; M1, left mastoid; M2, right mastoid; PSG#, polysomnography recording; REM, rapid eye movement sleep; SWS, slow-wave sleep. Patients spent significantly less time in SWS compared to the control group (^∗^p = 0.016; see inset for a magnified view of the group difference in SWS). On the top right, we report the electrode layout we used for the EEG (STAR Methods). Scoring of sleep stages was based on the current American Academy of Sleep Medicine scoring rules. (B) Cumulative percentages of time spent in each sleep stage are shown and highlight the low variability across the 3 nights in both groups. Specifically, SWS was not different across the 3 nights for patients (Friedman statistic = 0.29; degrees of freedon [df] = 2; p = 0.87) or for the controls (Friedman statistic = 0.67; df = 2; p = 0.72). White arrows indicate the percentage of SWS on each night for the controls, whereas SWS was significantly reduced in the patients.

Sleep is traditionally divided into different stages, each with defining patterns of EEG activity—non-rapid eye movement (NREM) sleep, which comprises stages N1, N2, and N3, with the latter also known as slow-wave sleep (SWS), and rapid eye movement (REM) sleep. We next compared the percentage of time, and also number of minutes, spent in each stage by the patients and controls (see Table 1 for summary data and statistical analyses; STAR Methods). Sleep measures were averaged across 3 nights (not including the habituation night). The time spent in N1, N2, and REM sleep was comparable between the groups. However, the patients spent significantly less time in SWS compared to the control participants—a mean of 1.31 min versus 18.02 min, respectively (Figure 1A). Moreover, this significantly reduced SWS was evident on each of the 3 nights when PSG was performed, with no statistical differences between the nights (Figure 1B). We also performed a bootstrapped analysis to assess group differences, and this confirmed a specific decrease in SWS in patients relative to the control participants (p = 0.020; 95% confidence interval [CI] [−7.85 to −2.37]; see Table S3). In an additional analysis, we examined the N2 and SWS data using an automatic slow-wave detection algorithm that was tailored to detect slow waves according to the sleep staging visual scoring rules [12, 13] (STAR Methods). This showed that the correspondence between the visual scoring by the sleep technologist and the automatic estimate of SWS was substantial (r = 0.814; p < 0.001). Therefore, selective bilateral hippocampal damage did not adversely impact the broad characteristics of sleep macroarchitecture; instead, the effect seemed to be confined to SWS. This suggests that SWS, which is held to be one neural signature of memory consolidation during sleep [14, 15], is facilitated by the hippocampus.

### Sleep Stability and Fragmentation

The apparently specific impact of hippocampal damage on SWS was further underlined by analyses of sleep stability and fragmentation (see Table 1 for summary data and statistical analyses; STAR Methods). Despite the reduced SWS in the patients, measures including the number of arousals/awakenings per hour of total sleep time, the number of shifts from one state to another, the number of periods of functional uncertainty [16], the number of shifts from any sleep stage to wakefulness, and the overall stability of NREM and REM sleep were not significantly different between the patients and control participants [17].

### Power Spectral Analyses

**Figure 2:**
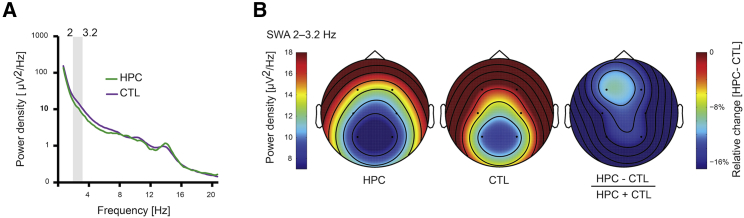
Power Spectra in N2 Sleep in the Patient and Control Participants (A) EEG power from 0.6–20 Hz for N2 sleep in patients (green line) and controls (purple line). This graph is based on the central EEG electrode C4. We used a data-driven bootstrapping approach to assess group differences in EEG power density spectra. Bootstrapped tests showed that the patients had reduced power in N2 delta activity compared to controls, from 2.0 to 3.2 Hz; p < 0.05 (indicated by gray shading on the graph). (B) Topographical head plots of EEG-quantified differences between patients and controls in slow-wave activity (SWA) (2–3.2 Hz) for C4. Cooler colors represent lower values. SWA is represented for patients in the left panel and for controls in the middle panel. The relative difference between the two groups (on the right) shows that the patients had decreased SWA (darker blue) compared to the control participants.

We next analyzed the PSG data using a quantitative, data-driven approach (STAR Methods). Bootstrapped power spectral density (PSD) analyses focusing on both N2 sleep and SWS showed a significant decrease in delta-band power in the EEG—the hallmark of SWS that, in the context of PSD, is known as slow-wave activity (SWA). This reduction in SWA was evident bilaterally at centro-parietal locations C3 (2.0–2.4 Hz; 2.8–3.2 Hz), C4 (1.8–4.0 Hz), P3 (2.8–3.6 Hz), and P4 (2.2–3.2 Hz). A similar result pertained when just N2 sleep was considered—C3 (2.2 Hz; 3.0 Hz), C4 (2.0–2.4 Hz; 2.8–3.2 Hz; Figure 2), and P4 (2.4–3.2 Hz). When we tested EEG PSD across the entire frequency range (0.6–20 Hz), we did not observe any other significant group differences. This alternative method of interrogating the PSG data, therefore, aligned with the visual sleep staging in revealing a specific alteration in SWA in the context of focal bilateral hippocampal damage.

### NREM Microarchitecture

We then tested whether any other features of N2 sleep might presage the decrease in SWS/SWA in the patients. We were particularly interested in slow oscillations (SOs) (0.5–1 Hz), slow (9–12 Hz) and fast (12–15 Hz) spindles, and the coupling of SOs and fast spindles, with the latter two features in particular thought to be markers of memory consolidation [6, 7, 18, 19, 20, 21].

**Figure 3:**
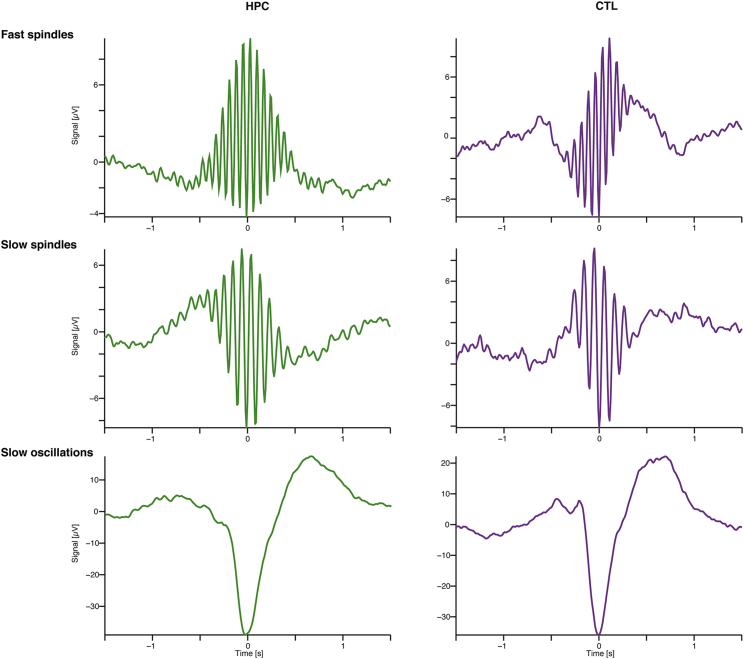
Example Sleep-Related Oscillations from a Patient and a Control Participant in N2 Sleep Here, we show averaged sleep-related oscillations for one patient (HPC, left panels, green lines) and one control participant (CTL, right panels, purple lines) for fast (upper panel) and slow (middle panel) sleep spindles as well as for slow oscillations (bottom panel) in N2 sleep with the events time-locked to the most negative trough (time = 0). These graphs demonstrate that characteristics of these sleep markers, such as their shape and amplitude, were similar between the two groups (STAR Methods; Data S1A and S1B).

We examined a number of SO properties—density, amplitude, duration, and slope—and those of spindle activity—density, amplitude, duration, and core frequency. We found no differences between the patients and control participants on any of these measures across frontal, central, and parietal electrodes (all MWUs p > 0.05; see summary data and statistics in Data S1A; Figure 3). We also performed a bootstrapped analysis (Data S1B). A trend, which indicated a potential decrease in SO density in patients (F4 density; p = 0.090; Data S1A), reached significance in the bootstrapped analysis (p = 0.029; 95% CI [−0.58 to −0.11]; Data S1B), suggesting that slow waves might not have sufficiently accumulated to 20% of the epoch, as required by visual staging of SWS (STAR Methods). Related to this finding, visual scoring of SWS was strongly associated with the density of slow waves during NREM stages, as measured by the automatic slow wave detection algorithm (r = 0.687; p = 0.007); this was not the case for slow-wave amplitude (r = −0.425; p = 0.130). Overall, these findings suggest that patients produced fewer slow waves, and this might explain the significantly reduced visually scored SWS.

Considering next the coupling of SO and fast spindles, the number of times these features were coupled was comparable in both groups (all MWUs p > 0.05; Data S1A and S1B). However, despite a similar number of couplings, fast spindles occurred later in the SO cycle in patients compared to the control participants, with a longer delay to the SO down-state (F3, MWU = 6, p = 0.048, Cohen’s *d* = 1.25; P3, MWU = 6, p = 0.048, Cohen’s *d* = 1.25; P4, MWU = 5, p = 0.034, Cohen’s *d* = 1.38; Data S2A). This was accompanied by a longer duration for SO that nested the delayed spindles in patients compared to the controls (F3, MWU = 6; p = 0.048; Cohen’s *d* = 1.25). These SO-fast spindle coupling effects were also apparent in a bootstrapped analysis (Data S2B).

## Discussion

Previous studies that have reported a reduction in SWS included patients with non-selective medial temporal lobe damage that also compromised other aspects of the sleep phenotype (e.g., [22]). Normal sleep architecture has been observed in patients with hippocampal sclerosis arising from chronic temporal lobe epilepsy, but this was in the context of a mild memory impairment [23]. By contrast, our novel contribution is a comprehensive analysis of the sleep phenotype in a small sample of four rare patients with both a significant episodic memory deficit and selective bilateral hippocampal damage. This revealed that, compared to matched control participants, the patients had significantly reduced SWS/SWA as measured by visual sleep staging, and a PSD analysis. Based on an automatic slow-wave detection algorithm, we also showed that the reduction in SWS in patients was likely due to decreased density, but not amplitude, of slow waves. In contrast, slow and fast spindles were indistinguishable from those of the control participants. Moreover, patients expressed SOs, and SO-fast spindle coupling was observed. However, we noted that SO density was reduced, with SOs that nested spindles having a longer duration, and the timing of spindles within the SO cycle was delayed in the patients. All other features of the sleep phenotype that we tested were similar between the groups.

The prevailing view of the sleep process holds that the interplay between three types of oscillations during NREM sleep facilitates a dialog between the hippocampus and neocortex that supports memory consolidation [18, 24, 25, 26, 27, 28, 29, 30, 31]. The reactivation of newly encoded memories is coordinated during the transition to cortical up-states after down-states marked by SOs (∼0.75 Hz). During this transition, starting around the SO down-state, thalamo-cortical spindles (9–15 Hz) [32, 33, 34, 35] are triggered that nest ripple activity (100–250 Hz) concurrently in cortical and hippocampal sites, and coincide with hippocampal-neocortical interactions. Typically, fast spindles, often synchronized to the transition, or early phase, of SO up-state depolarization [36], are associated with increased connectivity between hippocampus and neocortex [37]. Moreover, the fine-tuning of such SO-fast spindle coupling is thought to be essential for memory consolidation during sleep [6, 7].

Hippocampal ripples and cortical ripple activity cannot be reliably measured non-invasively in humans, but cortical SO and spindles that time-lock and quantify them are detectable using PSG. Given that our patients had significantly reduced SWS, we could not examine oscillations in this context. Instead, we focused on their N2 sleep to ascertain whether the oscillations helped to elucidate the nature of the reduced SWS, and hint at what role the hippocampus might play in the sleep process.

Slow and fast spindles were readily apparent in the patients’ N2 sleep and in the expected topology, and were indistinguishable from those of the control participants across a range of properties. Although patients expressed SOs, and SO-fast spindle coupling was observed, these sleep features were not sufficient to support normal memory function, likely because of some intriguing group differences that were apparent on closer scrutiny. The timing of spindles within the SO cycle was delayed in the patients. Cortical spindles and hippocampal ripples can be concurrent just after the down-state and in the transition to the cortical up-state within SO [31]. The shift of patients’ spindles into the later phase of the up-state within the SO cycle may indicate a mismatch in timing across the tripartite SO-spindle-ripple events that are critical for memory consolidation.

This shift might also explain the protracted activity (longer duration) of the SOs that nested the delayed spindles in patients compared to controls. Importantly, SOs were longer only when they nested spindles; otherwise, they seemed comparable to those of the control participants (Data S1A and S1B). This protracted activity within SOs might have hindered the initiation of another SO down-state, reducing SO density and, consequently, SWS. Studies examining the relationship between hippocampal activity and cortical SWS oscillations have demonstrated correlations between hippocampal function and up- and down-states in the cortex [38, 39]. Here, we show that, after focal lesions to the hippocampus, a reduction in SWS was observed, which was further characterized by decreased underlying SWA and SO density, and alterations in the coordination of memory-supporting spindle activity during SOs. This suggests that the hippocampus might mediate these associations and fine-tune the timing of cortical events, a finding that has not been clear from the previous correlational data alone.

SWA is generated from large populations of cortical neurons firing in synchrony, with modulation of these oscillations occurring via GABA interneurons, intrinsic network activity, and thalamic triggering of the up-states [40, 41]. SWA has been suggested to originate mainly from the medial frontal cortex [42]. Consequently, a key question for future research is to establish precisely how the hippocampus has such a profound effect on large-scale oscillatory activity in the cortex. This should include examining whether the hippocampus serves to modulate the inhibitory interneurons that mediate the generation of SWA, as a critical trigger for the up-state of the SO, or as a catalyst for activity-driven homeostatic control of these oscillations.

In summary, across a number of concordant analyses, our results revealed that focal bilateral hippocampal damage was associated with a fracture of sleep architecture in the form of reduced SWS and SWA, along with an apparent mistiming of fast spindles at the end of the SO cycle. This was despite an otherwise striking degree of preservation of the sleep phenotype. This suggests that, as with awake cognition, the hippocampus plays a significant role in sleep physiology that may in turn be necessary for the proper functioning of episodic memory.

## STAR★Methods

### Key Resources Table

**Table:** 

REAGENT or RESOURCE	SOURCE	IDENTIFIER
**Software and Algorithms**		

SpiSOP tool	RRID: SCR_015673	https://www.spisop.org
SleepTrip toolbox	RRID: SCR_017318	https://www.sleeptrip.org
MATLAB 2013b Mathworks	RRID: SCR_001622	https://uk.mathworks.com/
FieldTrip Toolbox [43]	RRID: SCR_004849	http://www.fieldtriptoolbox.org/
IBM SPSS Statistics 25	RRID: SCR_002865	https://www.ibm.com/analytics/us/en/technology/spss/
BrainVision Analyzer 2	RRID: SCR_002356	https://www.brainproducts.com/productdetails.php?id=17&tab=2
ProFusion PSG	Compumedics	https://www.compumedics.com.au/products/profusion-sleep-software/
zzzPAT software (version 4.4.64.p)	Itamar Medical Ltd., Caesarea, Israel	https://www.itamar-medical.com/unifiedupgrade/
Respironics Actiware	RRID: SCR_016440	http://www.actigraphy.com/solutions/actiware/

### Lead Contact and Materials Availability

Further information and requests for resources should be directed to and will be fulfilled by the Lead Contact, Eleanor Maguire (e.maguire@ucl.ac.uk). This study did not generate new unique reagents.

### Experimental Model and Subject Details

For all patients, hippocampal lesions resulted from voltage-gated potassium channel complex antibody-mediated limbic encephalitis (LGI1 VGKC-complex LE) [44]. This sleep study was conducted a median of 9.5 years after hippocampal damage occurred (mean 9 years ± SD 2.45). Patients (HPC) and the sleep control participants (CTL) were closely matched on a number of demographic factors: gender (all males), age (MWU = 19.00, p = 0.89, Cohen’s *d* = 0.08), body mass index (HPC mean 27.68 ± 2.51; CTL 25.79 ± 2.41; MWU = 14.00, p = 0.40, Cohen’s *d* = 0.47) and general cognitive ability assessed with the Matrix Reasoning subtest of the Wechsler Abbreviated Scale of Intelligence (WASI [45];) (MWU = 7.00, p = 0.06, Cohen’s *d* = 1.13). Age-related changes in sleep are well documented, with SWS typically reduced in older compared to young adults [46]. The time spent in SWS by our healthy control participants was comparable to that reported in the literature for older, male adults [47, 48], yet, importantly, was significantly greater than that of the patients to whom they were matched. All participants gave written informed consent to participate in accordance with the University College London research ethics committee.

The patients entered the sleep study having already been characterized, relative to matched healthy control participants, in terms of their lesion selectivity and neuropsychological profile as part of previous research studies. Full details of that characterization process are available here [49, 50, 51]. In summary, manual (blinded) segmentation of the hippocampi from T2-weighted high resolution structural MRI scans (0.5 × 0.5 × 0.5 mm voxels) showed that our patients (n = 4) had substantial volume loss relative to controls (n = 11) in the left (HPC mean 2417.00 mm^3^ ± 472.36; CTL 3173.18 mm^3^ ± 338.89; MWU = 2.00, p = 0.009, Cohen’s *d* = 1.83) and right (HPC 2515.00 mm^3^ ± 545.15; CTL 3285.91 mm^3^ ± 300.81; MWU = 3.00, p = 0.013, Cohen’s *d* = 1.67) hippocampus (Figure S1; Table S1). Expert neuroradiological examination confirmed there was no damage outside of the hippocampi. In addition, automated whole brain voxel-based morphometry showed there were no volume differences between patients and controls anywhere else in the brain. Table S2 provides the neuropsychological profile (summary data and statistical analyses) of the patients across a range of cognitive tests, and indicates the selective nature of their memory loss.

Since all patients included in the current study had suffered from LGI1 VGKC-complex antibody LE, our findings might potentially not generalize to other forms of hippocampal amnesia. However, it is important to note that other etiologies that lead to hippocampal-mediated amnesia such as viral encephalitis, hypoxic brain injury secondary to drug overdose, or toxic shock syndrome are associated with circumscribed hippocampal lesions, but frequently also involve anatomical damage elsewhere [52, 53]. In addition, these etiologies lead to co-morbidities and broader cognitive impairment [52, 54, 55, 56, 57, 58], which were absent from the clinical and neuropsychological profile of the patients reported here. Therefore, the selection of such a rare group of patients with circumscribed hippocampal lesions allowed us to pinpoint the direct role of the hippocampus in sleep physiology without the interference of potential confounds associated with heterogeneity in etiology.

Other features associated with LGI1-antibody LE in its initial presentation – such as focal seizures and hyponatremia related to hypothalamic damage – are also unlikely to explain the effects we observed. Our patients were seizure-free when they were discharged after initial admission, they were not prescribed antiepileptic medication, and none of the patients had seizure recurrence following initial treatment. Thus, unlike in temporal lobe epilepsy, which is associated with ongoing seizures and hippocampal sclerosis [59], our patients enabled us to study effects on SWS that were not coincidental with, and sequelae of, seizure activity. Moreover, patients were not undergoing treatment for hyponatremia, which is consistent with published evidence that persistent hyponatremia is not a characteristic feature of LGI1-antibody LE [60]. Crucially, there are no published studies that report lesions in the hypothalamus. Therefore, the findings in the current study are unlikely to stem from the above-mentioned potential issues.

### Method Details

#### Equipment

The WatchPAT-200 (Itamar Medical Ltd., Caesarea, Israel) is a wrist-worn device designed to assess the severity of obstructive sleep apnoea syndrome (OSA) by measuring the Peripheral Arterial Tone (PAT) signal by means of a plethysmographic based finger-mounted probe. This device was used during the habituation PSG night. Signals were automatically analyzed with the zzzPAT software (version 4.4.64.p, Itamar Medical Ltd., Caesarea, Israel) to identify respiratory events and sleep states. The outcome measure employed in this study was the PAT apnoea hypopnea index (AHI), which provides the number of apnoea and hypopnea events per hour during the night. We observed severe obstructive sleep apnoea (AHI > 30) in one patient. We, therefore, ensured that two of the participants in the control group were closely matched to this patient on AHI, gender, BMI, age, and general cognitive ability. Of note, when we conducted the analyses without this patient and his two matched controls, the results were unchanged (see Tables S4 and S5).

In order to assess sleep-wake patterns, participants wore an Actiwatch 2 (Phillips Respironics Mini-Mitter) for seven consecutive days and nights on their non-dominant wrist. Light and activity data were collected in 30 s epochs and analyzed using the Philips Actiware 6.0.2 software package (Respironics Actiware 6.0.2; RRID: SCR_016440). Data were scored based on available guidelines [61], with a medium sensitivity (40 activity cpm), with sleep onset occurring after an immobility period of 10 minutes, and rise time following an increase in activity level and in light level above 1.0 μW/cm^2^. Daily reminders to report time in a sleep diary (the Official Sleep Diary from the National Sleep Foundation) were sent to all participants via texts or phone calls in the morning upon awakening and in the evening before going to sleep. All participants completed the sleep diary, which was used to assist in scoring actigraphy. Because self-reported sleep duration is often not representative of the actual sleep period when assessed with concurrent objective sleep measures [62], if bedtime and rise time in the diary did not match with the actigraphy data, we used objective measures such as activity count, event marker and light level, in line with Chow et al. [61]. Variables of interest were sleep efficiency (in percent), total sleep time (in minutes), sleep fragmentation index (FI; an index of restlessness), night-to-night variability for sleep duration [63], average bedtime and mean sleep midpoint (clock time halfway between the bedtime and rise time).

Participants were allowed to sleep according to their habitual schedule (their usual bedtime and rise time), but were required to keep a stable sleep pattern for the duration of the study. They were also instructed to abstain from all caffeinated beverages after midday throughout the study. In addition, spouses/family members were asked to monitor the participants and maintain a stable sleep schedule. There were no differences between the patients and controls in meeting these requirements. Moreover, given that the majority of the participants were older adults, lifestyle factors that might influence the sleep schedule (e.g., busy work/family activities) were reduced compared to young adults.

All participants underwent PSG in their homes using a Brain Products system (GmbH, Gilching, Germany). On each night that PSG was recorded, two trained research technicians arrived at a participant’s home approximately three hours before the usual bedtime to set up for the PSG. Equipment was then removed by a research technician the following morning upon awakening. PSG was recorded using a 24-electrode cap (EasyCap; based on the international 10-20 system) including the following EEG channels: Fp1, Fp2, F3, F4, C3, C4, P3, P4, O1, O2, F7, F8, T7, T8, P7, P8, Fz, Cz, Pz, Oz, FT9, FT10 referenced to average mastoids (M1 and M2) (sampling rate = 500 Hz). This montage also included two bipolar electrooculogram channels (EOG), two electromyogram channels (EMG) and two electrocardiogram channels (ECG).

#### PSG Scoring

Sleep staging was performed by a registered polysomnographic technologist, who was blind to group membership and the study aims, based on EOG, EMG and the following derivations: F3/M2, F4/M1, C3/M2, C4/M1, O1/M2, O2/M1. Visual scoring of the recordings followed the current, widely-used American Academy of Sleep Medicine scoring rules (AASM) [64]. This includes the requirement for slow waves to occupy at least 20% of a 30 s epoch in order to be classified as SWS. To assess intra-rater variability, 20% of the polysomnographic data were blindly re-scored by the same sleep technologist with an interval of > 5 months between the first and second sleep stage scoring. Agreement based on quantitative sleep parameters derived from the sleep stage scoring was assessed by means of intra-class correlation (ICC) coefficients, with a 2-way mixed-effects model focusing on absolute agreement. This indicated moderate to excellent reliability [65]: total sleep time 0.9; REM 0.8; N1 0.9; N2 0.6; N3 0.9 and wake after sleep onset 0.9.

We also performed additional analyses to calculate power spectral density (PSD) for NREM (N2 and SWS), as well as N2 slow spindles, fast spindles, slow oscillations (SO), and SO-fast spindle coupling using the SpiSOP tool (https://www.spisop.org; RRID: SCR_015673), run in MATLAB 2013b (Mathworks, Natick, USA; RRID: SCR_001622). Sleep topoplots and event-related potentials were created using SleepTrip (https://www.sleeptrip.org; RRID: SCR_017318) based on FieldTrip functions (http://fieldtriptoolbox.org; RRID: SCR_004849 [43]) – template codes for these operations will be made available upon publication. Electrode sites were linked to the average potential from channels attached to the mastoids (M1, M2) with ground Fpz. Signals were then amplified (sampling rate > 250 Hz) and filtered (EEG and EOG 0.3-35 Hz, EMG 10-100 Hz). We focused on the following channels: F3, F4, C3, C4, P3, and P4. PSD was calculated by averaging fast Fourier transformation coefficients on Hanning-windowed consecutive 5 s intervals (i.e., 0.2 Hz resolution, using Welch’s method) of artifact-free NREM sleep (N2 and SWS), which overlapped in time by 4 s. PSD values were obtained by normalizing the power values by effective noise bandwidth. Epochs with EMG and EEG artifacts were excluded automatically from all the analyses. A human scorer checked the validity of the artifact detection. We examined group differences in EEG power density spectra across the entire frequency range (0.6-20 Hz).

All parameters for PSD, spindles, and SO were as reported in Wang et al. [66]. Parameters for SO-fast spindle coupling were as described by Thürer et al. [67], with the exception that SO were identified with a factor of 1.25 for the means of the amplitude and the negative half-wave peak potential, and only one threshold of 1.5 standard deviations of the filtered signal to mark spindles. Note that the SO detection targeted a frequency range of 0.5–1.11 Hz with resulting core frequencies of ∼0.75 Hz which are known to be coupled with fast sleep spindles (i.e., the NREM-typical slow waves of larger amplitudes). In brief, we identified SO that had at least one detected sleep spindle from the lowest trough (down-state) to +0.5 s after the next positive-to-negative zero crossing (i.e., slow wave up-state). Sleep spindles were counted only once for the first slow wave in which they occurred within the same channel. The mean delay of sleep spindles to the SO down-state and the standard deviation of this delay were calculated to estimate the temporal dispersion of their co-occurrence (delay dispersion). In addition, the average amplitude and duration of coupled SO and fast spindles were calculated. Epochs with EMG and EEG artifacts were excluded automatically from all automatic analyses. Therefore, whenever the EMG signal (filtered from 10-80 Hz) was not within a −50 to +50 μV range or the EEG signal (filtered from 0.3-33 Hz) was not in a −300 to +300 μV range, the epochs within −3 to +3 s of this incidence were automatically excluded as artifacts.

In an additional analysis we detected slow waves by tailoring the automatic detection algorithm to match the AASM scoring definition, i.e., durations corresponding to 0.5-2 Hz, amplitudes of 75-400 μV and detection averaged over F3/M2, F4/M1, C3/M2, C4/M1. Furthermore, we excluded slow waves with durations, frequencies or amplitudes exceeding 3 standard deviations from their respective mean in each channel. We then summed the durations of all detected slow waves in each visually scored N2 and SWS epoch. If the summed duration in an epoch exceeded 6 s (i.e., 20% of the 30 s epoch), then this epoch was classified as an epoch estimating SWS.

### Quantification and Statistical Analysis

All statistical analyses were performed with SPSS 25.0 (IBM Corporation; RRID: SCR_002865). Given that the data did not meet the assumptions of normality and homogeneity necessary for parametric statistics, the majority of the between-group analyses were performed using non-parametric Mann-Whitney U tests. Group differences in the PSD analyses were examined using bootstrapped independent-sample t tests [68, 69]. In all analyses, the significance level was set at 0.05. Given the small number of participants, we also included confidence intervals and effect size estimates using non-parametric Cohen’s *d* for all outcome variables. In addition, we performed bootstrapping (sampling the dataset with resampling; 1000 iterations) to assess group differences on all sleep measures (Data S1B and S2B; Table S3).

### Data and Code Availability

The data are available upon request by contacting the Lead Contact, Eleanor Maguire (e.maguire@ucl.ac.uk).

## Supplemental Information

Document S1. Figure S1 and Table S1

Table S2. Summary of Neuropsychological Information, Related to the Experimental Model and Subject Details in the STAR Methods M = mean; SD = standard deviation; 95% CI = 95% confidence interval; ES = effect size; HPC = hippocampal-damaged patients; CTL = control participants; WASI = Wechsler Abbreviated Scale of Intelligence; WMS = Wechsler Memory Scale III; DKEFS = Delis-Kaplan Executive Function System; VOSP = Visual Object and Space Perception Battery; HADS = Hospital Anxiety and Depression Scale. For the Autobiographical Interview, the scores are the average number of internal (or external) details over five memories. ^a^The control group consisted of twelve participants (all males, mean age 57.2 years ± 16.6). ^b^The control group consisted of eight participants (six males, mean age 54.63 years ± 16.32). P values relate to between-group non-parametric Mann-Whitney U tests with significant differences depicted in bold.

Table S3. Sleep Characteristics of the Patients and Controls with a Bootstrapping Procedure, Related to Table 1 95% CI = 95% confidence interval; NREM = non-rapid eye movement sleep; REM = rapid eye movement sleep; SWS = slow-wave sleep; min = minute; TFU = total functional uncertainty; TST = total sleep time. Sleep Macroarchitecture and Sleep Stability and Fragmentation measures are averaged over three nights. P values relate to between-group analyses based on a bootstrapping procedure with significant differences depicted in bold.

Table S4. Sleep Characteristics of the Patients and Controls without the OSA Participants, Related to Table 1 OSA = obstructive sleep apnoea; M = mean; SD = standard deviation; 95% CI = 95% confidence interval; ES = effect size; HPC = hippocampal-damaged patients; CTL = control participants; PSQI = Pittsburgh Sleep Quality Index; ESS = Epworth Sleepiness Scale; MEQ = Morningness-Eveningness Questionnaire; NREM = non-rapid eye movement sleep; REM = rapid eye movement sleep; SWS = slow-wave sleep; min = minute; TFU = total functional uncertainty; TST = total sleep time. Sleep Macroarchitecture and Sleep Stability and Fragmentation measures are averaged over three nights. P values relate to between-group non-parametric Mann-Whitney U tests with significant differences depicted in bold.

Table S5. Characterization of Spindles, Slow Oscillations, and SO-Fast Spindle Coupling at Frontal, Central, and Parietal Sites for the Patients and Controls in N2 Sleep without the OSA Participants, Related to Method Details in the STAR Methods OSA = obstructive sleep apnoea; M = mean for each group; SD = standard deviation; 95% CI = 95% confidence Interval; ES = effect size; HPC = hippocampal-damaged patients; CTL = control participants; min = minute; s = second; epoch = 30 s. P values relate to between-group non-parametric Mann-Whitney U tests.

Data S1. Characterization of Spindles, Slow Oscillations, and SO-Fast Spindle Coupling at Frontal, Central, and Parietal Sites for the Patients and Controls in N2 Sleep, Related to Method Details in the STAR Methods (A) M = mean for each group; SD = standard deviation; 95% CI = 95% confidence interval; ES = effect size; HPC = hippocampal-damaged patients; CTL = control participants; min = minute; s = second; epoch = 30 s. P values relate to between-group non-parametric Mann-Whitney U tests. (B) P values relate to between-group analyses based on a bootstrapping procedure with significant differences depicted in bold.

Data S2. Further Characterization of SO-Fast Spindle Coupling at Frontal, Central, and Parietal Sites for the Patients and Controls, Related to Method Details in the STAR Methods (A) M = mean for each group; SD = standard deviation; 95% CI = 95% confidence interval; ES = effect size; HPC = hippocampal-damaged patients; CTL = control participants; SO = slow oscillations; s = seconds. P values relate to between-group non-parametric Mann-Whitney U tests with significant differences depicted in bold. (B) P values relate to between-group analyses based on a bootstrapping procedure with significant differences depicted in bold.

Document S2. Article plus Supplemental Information
