# Supplementary material for: Sleeping with Hippocampal Damage
Source: Curr Biol. 2020 Feb 3;30(3):523–529.e3. doi: 10.1016/j.cub.2019.11.072 (PMC6997880; doi:10.1016/j.cub.2019.11.072)
Supplement: Document S1. Figure S1 and Table S1 [file mmc1.pdf]

Current Biology, Volume *30*

Supplemental Information

Sleeping with Hippocampal Damage

Goffredina Spanò, Frederik D. Weber, Gloria Pizzamiglio, Cornelia McCormick, Thomas D. Miller, Clive R. Rosenthal, Jamie O. Edgin, and Eleanor A. Maguire

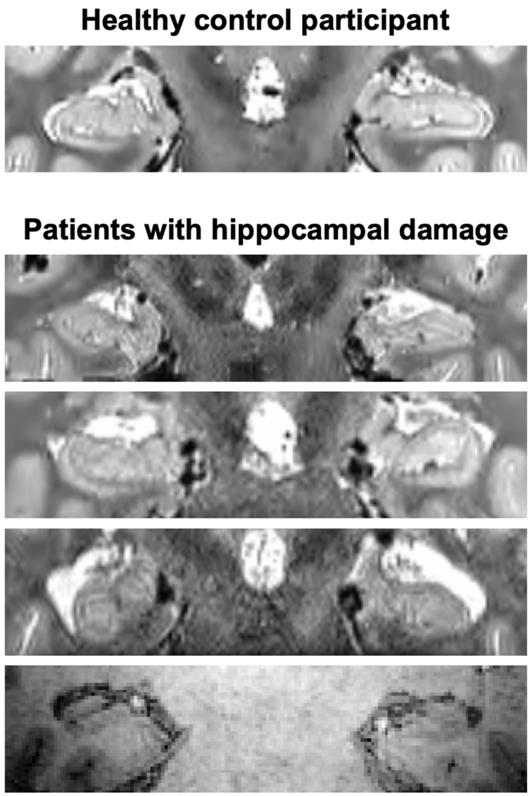

Figure S1. MR images from one example control participant and each of the four patients. Related to the Experimental Model and Participant Details in the STAR Methods. For the control participants and the top 3 patient panels, we show T2-weighted structural MR images (0.5mm isotropic voxels). For one patient (bottom panel), only a T1-weighted image (1mm isotropic voxels) was available. Images are displayed in native space corresponding approximately to the position of y = -10 in the MNI coordinate system. Note the dilatation of the temporal horns that is associated with the hippocampal atrophy. As shown here and from the percentage volume loss in Table S1, patients suffered from selective bilateral hippocampal damage.

Group	Age	Chronicity	LHPC volume (mm^3)	RHPC volume (mm^3)	LHPC % volume loss relative to CTL[a]	RHPC % volume loss relative to CTL[a]	WASI	SWS%
CTL	59.20 (15.89)	n.a.	3173.18[a] (338.89)	3285.91[a] (300.81)	n.a.	n.a.	14.50 (2.37)	5.39 (4.57)
HPC1	61	6	2506	2803	-21.03%	-14.70%	12	0.10
HPC2	72	8	1736	1698	-45.29%	-48.32%	10	0.00
HPC3	72	11	2607	2755	-17.84%	-16.16%	12	0.27
HPC4	28	11	2819	2804	-11.16%	-14.67%	14	0.97

Table S1. Summary of demographic characteristics and SWS. Related to the Experimental Model and Participant Details in the STAR Methods. All control participants and patients were right-handed males. Mean and standard deviation in parentheses are shown for control participants and individual data for the 4 patients. Age and chronicity are expressed in years. [a]The control group consisted of eleven participants (mean age 55.64 years ± 16.47). LHPC = left hippocampus; RHPC = right hippocampus; WASI = Wechsler Abbreviated Scale of Intelligence [S1] Matrix Reasoning subtest scaled score; SWS = slow-wave sleep.

Supplemental References

S1. Wechsler, D. (1999). Wechsler Abbreviated Scale of Intelligence (New York, NY: The Psychological Corporation, Harcourt Brace).

S2. Levine, B., Svoboda, E., Hay, J.F., Winocur, G., and Moscovitch, M. (2002). Aging and autobiographical memory: Dissociating episodic from semantic retrieval. Psychol. Aging *17*, 677–689.

S3. Wechsler, D. (1997). The Wechsler Memory Scale, Third Edition (San Antonio, TX: The Psychological Corporation).

S4. Osterrieth, P.A. (1944). The test of copying a complex figure: a contribution to the study of perception and memory. Arch. Psychol. (Geneve). *30*, 286 –356.

S5. Warrington, E.K. (1984). Recognition Memory Test: Manual (Berkshire, UK: NFER-Nelson).

S6. McKenna, P., Warrington, E.K. (1980). Testing for nominal dysphasia. J. Neurol. Neurosurg. Psychiatry *43*, 781–788.

S7. Warrington, E.K. (2010). The graded naming test: a restandardisation. Neuropsychol. Rehabil. *7*, 143–146.

S8. Delis, D.C., Kaplan, E., Kramer, J.H. (2001). Delis Kaplan Executive Function System (D-KEFS) (San Antonio, TX: The Psychological Corporation).

S9. Burgess, P., Shallice, T. (1997). The Hayling and Brixton tests. Test Manual. (Bury St Edmunds, UK: Thames Valley Test Company).

S10. Warrington, E.K., James, M. (1991). A new test of object decision: 2D silhouettes featuring a minimal view. Cortex *27*, 370 –383.

S11. Gabrovska, V., Laws, K., McKenna, P. (1996). Visual form perception in schizophrenia: further evidence for a disorder of semantic memory. Eur. Psychiatry *11*, 278.

S12. Zigmond, A.S., Snaith, R.P. (1983). The hospital anxiety and depression scale. Acta Psychiatr. Scand. *67*, 361–370.
